# Feeding type and development drive the ingestion of microplastics by freshwater invertebrates

**DOI:** 10.1038/s41598-017-17191-7

**Published:** 2017-12-05

**Authors:** Christian Scherer, Nicole Brennholt, Georg Reifferscheid, Martin Wagner

**Affiliations:** 1 Goethe University Frankfurt/Main, Department Aquatic Ecotoxicology, Max-von-Laue-Straße 13, D-60323 Frankfurt/Main, Germany; 2 Federal Institute of Hydrology, Department Biochemistry and Ecotoxicology, Am Mainzer Tor 1, D-56002 Koblenz, Germany; 3 Norwegian University of Science and Technology, Department of Biology, Høgskoleringen 5, Realfagbygget, NO-7491 Trondheim Norway

## Abstract

Microscopic plastic items (microplastics) are ubiquitously present in aquatic ecosystems. With decreasing size their availability and potential to accumulate throughout food webs increase. However, little is known on the uptake of microplastics by freshwater invertebrates. To address this, we exposed species with different feeding strategies to 1, 10 and 90 µm fluorescent polystyrene spheres (3–3 000 particles mL^−1^). Additionally, we investigated how developmental stages and a co-exposure to natural particles (e.g., food) modulate microplastic ingestion. All species ingested microplastics in a concentration-dependent manner with *Daphnia magna* consuming up to 6 180 particles h^−1^, followed by *Chironomus riparius* (226 particles h^−1^), *Physella acuta* (118 particles h^−1^), *Gammarus pulex* (10 particles h^−1^) and *Lumbriculus variegatus* (8 particles h^−1^). *D. magna* did not ingest 90 µm microplastics whereas the other species preferred larger microplastics over 1 µm in size. In *C. riparius* and *D. magna*, size preference depended on the life stage with larger specimens ingesting more and larger microplastics. The presence of natural particles generally reduced the microplastics uptake. Our results demonstrate that freshwater invertebrates have the capacity to ingest microplastics. However, the quantity of uptake depends on their feeding type and morphology as well as on the availability of microplastics.

## Introduction

Since the 1950s plastic materials have basically permeated everyday life. Besides massive economic and social benefits of synthetic polymers, unsustainable resource management has resulted in plastic materials entering the environment. Here, their main advantages (e.g., durability, lightweight) promote the accumulation and mobility in ecosystems. When exposed to abiotic and biotic weathering processes, polymers fragment to increasingly smaller particles^1,2^ referred to as microplastics^3^. Over the last decade, numerous studies have reported the ubiquitous presence of microplastics in surface waters and sediments. Primarily focused on marine systems, an increasing number of recent studies demonstrate that freshwater compartments are contaminated to a comparable degree^4–6^.

Owing to their small size, microplastics are potentially available for a broad range of aquatic species. Indeed, microplastic ingestion has been shown for several field collected marine and freshwater species^7–9^. Additionally, ecologists have frequently used polymer beads to investigate the feeding behavior of pelagic freshwater zooplankton. In their attempt to categorize filtering capacities of different species and their ability to feed on bacteria and algae, they collected essential data for a better understanding of community assemblages in various habitats^10,11^.

Interestingly, 20–30 years later, microplastics re-emerge as pollutant of emerging concern. Laboratory studies with invertebrates documented their adverse effects across various taxa, including reduced feeding, weight and fertility as well as induced mortality and inflammation^12–15^. The overwhelming majority of toxicity studies is available on marine species and adverse effects has been observed for copepods^12,14^, the lugworm *Arenicola marina* ^15^ and the blue mussel *Mytilus edulis* ^13^. In contrast, other studies report that microplastics exposure does not induce toxicity, for instance in the isopod *Idotea emarginata* ^16^ and the larvae of the sea urchin *Tripneustes gratilla* ^17^.

Compared to marine species, uptake and toxicity data for freshwater biota are scarce. The capacity of several freshwater invertebrates to ingest microplastics has already been reported in a quantitative approach to assess filtration rates of pelagic filter feeders^18–20^ and in a qualitative approach of different feeding types^21^. Only few studies documented adverse effects on freshwater organisms by microplastic exposure including the waterflea *Daphnia magna* ^22–24^, the amphipod *Hyalella azteka* ^25^ and the fish *Danio rerio* ^26^, whereas the freshwater snail *Potamopyrgus antipodarum* was not affected throughout their development^27^.

These interspecific differences highlight the complex interactions of organisms and microplastics. Accordingly, it is crucial to understand the factors determining the outcome of microplastic exposure. Ingestion of microplastics and, thus, internal exposure are the prerequisites to induce toxicity. In general, factors affecting feeding are manifold and include abiotic (e.g., temperature) and biotic factors (e.g. appetite of individuals, food concentration, taste). Additionally, the ingestion rates differ between species and feeding strategies (e.g., filter *vs* deposit feeders). While these factors have been investigated for natural feeding regimes, it is so far unknown what drives the ingestion of microplastics by freshwater invertebrates.

Thus, we investigated the feeding on microplastics by the water flea *Daphnia magna* (filter feeder), the aquatic larvae of the diptera *Chironomus riparius* (collector-gatherer), the oligochaete *Lumbriculus variegatus* (deposit feeder), the amphipod *Gammarus pulex* (shredder) and the snail *Physella acuta* (scraper and surface grazer) under laboratory conditions. To evaluate the uptake of microplastics by the five different feeding types quantitatively, we investigated the factors particle size (1, 10 and 90 µm in diameter) and particle concentration (3–3 000 P mL^−1^). Therefore, we exposed the organisms to fluorescent polystyrene spheres in several species-specific short-term exposure regimes. To include more realistic parameters, we also investigated the co-exposure to additional natural particles (algae, sand and leaf) as well as different developmental stages of *C. riparius* and *D. magna*. This study aims at a better understanding of the (a)biotic factors affecting the ingestion of microplastics by freshwater invertebrates and, thus, provide important data for future toxicity testing.

## Results and Discussion

### Concentration

**Figure 1:**
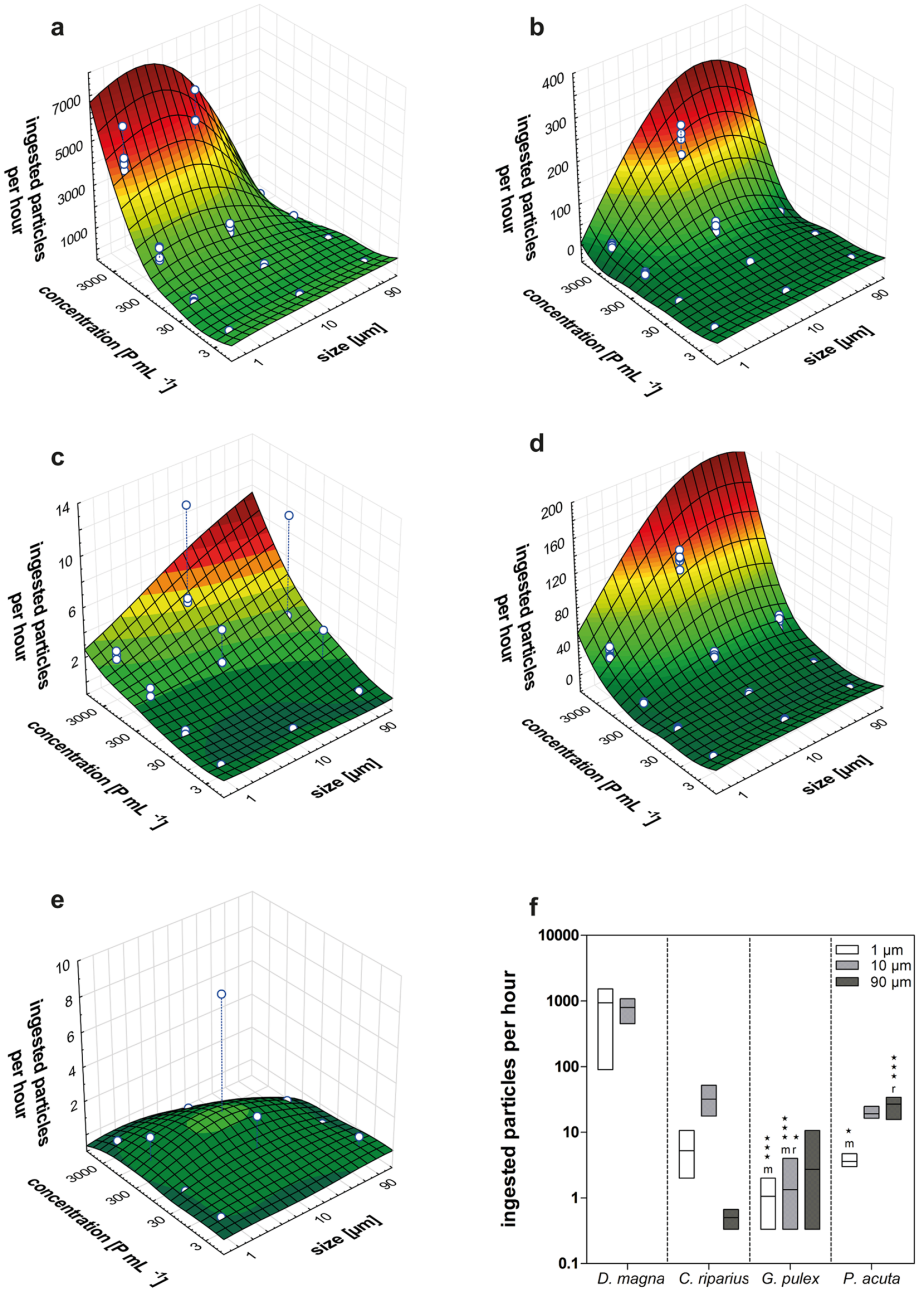
Feeding on polystyrene particles. (**a**–**e**) Surface plots of the amount of ingested particles (1, 10 and 90 µm) per hour by *Daphnia magna* (**a**), *Chironomus riparius* (**b**), *Gammarus pulex* (**c**), *Physella acuta* (**d**) and *Lumbriculus variegatus* (**e**) at different particle concentrations (3–3 000 P mL^−1^). n = 6. (**f**) Floating bars (min to max, line at mean) illustrating the number of ingested particles (1, 10 and 90 µm) per hour by *D. magna*, *C. riparius*, *G. pulex* and *P. acuta* exposed to 300 P mL^−1^. The lower case letters refer to statistical significant differences between the organism and *D. magna* (letter m) and *C. riparius* (letter r) at same particle size. *p < 0.05, ***p < 0.001. n = 6.

Our results show that the concentration of plastic particles affects the ingestion rates in all species (except *L. variegatus*). With increasing particle concentration, the proportion of bead-containing organisms as well as the number of ingested particles per individual increased (Fig. 1, Supplementary Table S2). Here, ingestion rates (P h^−1^) were significantly different between the lower and higher concentrations (p < 0.05, Supplementary Table S1). Presuming non-selective feeding, a high concentration leads to a high encounter rate resulting in an increased feeding rate. Furthermore, the likelihood to detect particles in the digestive tract of organisms at low concentrations is much lower compared to high concentrations. Thus, it may explain the overall low amount of bead containing organisms at concentrations of 3–30 P mL^−1^. Overall, the relation between feeding rate and concentration of food particles or tracers is in accordance with literature^16,28–32^. For instance, the feeding rates of daphnids increased linearly with food concentrations until it reached a plateau at high particle densities (>10^6^ P mL^−1^)^31^. Therefore, we can assume a quite similar response of our species, i.e., ingestion rates will steadily increase with particle concentration until the species reached their maximum feeding capacity.

### Size

All species ingested 1, 10, and 90 µm polystyrene beads, with the exception of *D. magna* which did not ingest 90 µm beads (Supplementary Table S2). Despite the intraspecific variability in feeding rates (Supplementary Table S2), uptake of 90 µm particles by *C. riparius* was significantly reduced compared to 10 µm particles (p < 0.01, Supplementary Table S1). Here, an upper size limit of particle ingestion might be determined by the head capsule morphology and is further discussed in the section on development-dependent ingestion.

In the case of *P. acuta*, the ingestion of 90 µm beads was significantly enhanced (p < 0.05, Supplementary Table S1) compared to the 1 µm particles. We did not expect a size-selective intake because *P. acuta* feeds on biofilms as scraper or surface grazer. Accordingly, the preference for larger particles might depend on the sedimentation behavior of the polystyrene spheres, which can be described using Stokes’ law. Because the density of the spheres (1.05 g cm^−3^) and the medium (1 g cm^−3^) was constant for all beads and experiments, sedimentation is determined by particle size (radius of the spheres) in our case. Accordingly, Stokes’ law predicts that 90 µm beads settle within seconds (0.02 cm s^−1^), 10 µm particles within minutes (0.016 cm min^−1^) and 1 µm particles within hours or even days (0.23 cm d^−1^). Although an exact prediction is complicated by the movement of the organisms resulting in a continuous mixing of the suspension, we visually observed a fast settling of 90 and 10 µm beads. Nevertheless, *P. acuta* had higher feeding rates for 10 and 90 µm particles compared to the 1 µm particles (p < 0.05, Fig. 1, Supplementary Table S2). This corroborates the assumption, that the larger particles sink faster and were, thus, more available for the benthic feeders *C. riparius*, *G. pulex*, *P. acuta* and *L. variegatus*.

Not only polymer density and particle size affect the sedimentation and ingestion process, but also the shape. We solely tested uniformly shaped spheres and did not address potential impacts of irregular shaped particles on feeding rates. Because the shape of particles affects the sedimentation process as well as the interaction with species, feeding rates and depuration of irregular shaped plastic particles or fibers will most likely differ^24,33^. In summary, concentration, density and size of plastic particles affect the exposure to and, thus, the uptake by aquatic organisms.

### Species

Besides particle properties determining the microplastics’ availability, the characteristics of the biological receptor drive the ingestion, too. In the absence of quantitative studies, we compared the microplastics uptake by freshwater invertebrates with different feeding strategies. Our results show clear differences in the ingestion rates between species (Fig. 1a–e). Comparing the ingestion rates at 300 P mL^−1^ (Fig. 1f), the patterns of the surface plots can be confirmed. While the pelagic *D. magna* preferably ingests smaller particles, the benthic feeder *C. riparius*, *G. pulex* and *P. acuta* preferably ingest particles in the upper size range. The lowest number of individuals containing polystyrene beads (6%) was detected in the benthic deposit feeder *L. variegatus* (Supplementary Table S2).

***L. variegatus*** is a globally distributed freshwater oligochaete, colonizing rivers, ponds and lakes^34^. Typically burrowing and feeding on subsurface sediments, the black worm ingests a mixture of organic (detritus, algae, bacteria) and inorganic material with particle sizes <100 µm^35,36^. Although we varied parameters like age and size of individuals, the exposure time and volume, the intraspecific variability remained high, suggesting that under the current exposure regime (i.e., absence of sediment) *L. variegatus* did not readily feed on microplastics. However, the examined specimens contained 1 µm (<5 beads), 10 µm (<24 beads) and 90 µm (<2 beads) polystyrene beads and a study by Beckingham and Ghosh^37^ documented the ingestion of 35 µm polypropylene beads demonstrating that the black worm has the capacity to ingest plastic particles of different sizes (Fig. 1, Supplementary Table S2).

***C. riparius*** builds vertical tubes within the top layer of sediments and feeds, as collector-gatherer, on detritus and its associated bacteria and fungi^38^. They are commonly seen projecting from their tubes or even completely outside of their tubes, apparently feeding on settled particulate matter. The chironomid gut contains a mixture of diatoms, detritus and a relatively high proportion of silt in a size range of 1 to 25 µm^39^, indicating a minor selectivity of feeding. This can be confirmed with regard to microplastics (Supplementary Table S2). In contrast to *L. variegatus*, *C. riparius* ingested polystyrene beads more regularly (66.6% of all tested individuals) and to a greater extent (up to 680 P (10 µm) per individual). This difference can be explained by the different feeding strategies. While *L. variegatus* feeds mainly burrowed in the substrate, the epibenthic chironomid larvae feed on the sediment surface. Therefore, an encounter and ingestion of settling plastic materials in their habitats seems likely. The ingestion rates point to a preference of 10 µm (<227 P h^−1^), followed by 1 µm (20.6 P h^−1^) and 90 µm (1 P h^−1^) beads (Fig. 1).

***G. pulex*** and other amphipod species represent a dominant macroinvertebrate group in riverine communities throughout Europe^40,41^. Commonly known as shredders (e.g., processing allochthonous leaf material) their feeding strategy is more complex. *G. pulex* can be classified as an herbivore, detrivore and predator^42^, implying that amphipods can ingest materials on the sediment surface, and throughout the water column. In our experiments, individual gammarids exposed to 10 and 90 µm beads contained up to 12 and 32 beads per individual (300 P mL^−1^), respectively. This implies a preference for larger microplastics (Fig. 1, Supplementary Table S2). The low amount of ingested 1 µm beads (Supplementary Table 2) is in accordance with former studies on amphipod species^43^. For instance, Blarer and Burkhardt-Holm^43^ found only two to seven 1.6 µm PS beads in 16 examined *Gammarus fossarum* specimens at exposure concentrations (500–2 500 P mL^−1^) similar to the ones used here. In contrast, the number of beads in the gut increased to > 100 at exposure concentrations of 12 500 and 60 000 P mL^−1^. Overall, the high intraspecific variability in feeding makes it difficult to derive consistent ingestion rates. Various factors like metabolic rates and physiological conditions seem to influence the appetite of individuals and consequently, their feeding behaviour^44^. Therefore, Agatz and Brown^45^ recommend conducting feeding studies with more than one individual per replicate (mass feeding) to reduce the intraspecific variability. Notwithstanding the inter-individual variability, our data demonstrates that *G. pulex* ingests settling and suspended plastic particles, confirming former studies on amphipod and isopod species^16,25,43^. Furthermore, their role as a predator and indeed as a prey may be an important factor considering the transfer of plastic particles throughout the trophic levels in freshwater communities.

***P. acuta*** is a globally distributed freshwater snail that colonizes streams, lakes and ponds^46,47^. As a scraper and surface grazer, it feeds on algae and detritus from biofilms. Accordingly, we expected a preferred ingestion of larger particles that settle faster. This was confirmed experimentally as *P. acuta* preferably ingested 10 and 90 µm particles with the highest feeding rate for the latter (Fig. 1, Supplementary Table S2). However, the snails also ingested 1 µm beads to a lesser extent. Assuming a non-selective feeding on the tested size-classes, the lower ingestion was mainly driven by the availability. Considering the long-term test duration of 24 h, the amount of settled 1 µm beads was higher compared to the short-term exposure of the other benthic species. A shorter exposure time, appropriate for a better species comparison, led to high intraspecific variability caused by variations in feeding activity (some individuals did not feed at all). Although we counted the egested particles, we might underestimate the amount of ingested particles per hour – because the re-ingestion of egested particles could not be excluded completely. *P. acuta* may preferably ingest settling plastic particles but their ability to graze on the water surface can also result in an encounter with buoyant polymers.

***D. magna*** is a pelagic filter feeder and exhibited the highest feeding rate on 1 and 10 µm beads compared to the other tested species, but did not ingest any 90 µm particle (Fig. 1f). The daphnids ingested significantly more beads per hour than *G. pulex* (890 × higher for 1 µm and 611× higher for 10 µm, Supplementary Table S1) and *P. acuta* (267 × higher for 1 µm, Supplementary Table S1). The essential role of daphnids in freshwater habitats is well described^48^. As a highly effective suspension feeder regulating algae and bacteria growth they occupy an important position in aquatic food webs. Mainly feeding on bacteria and algae, *D. magna* additionally ingest a wide range of seston components, including digestible and non-digestible particulate matter. The ingestion is mainly driven by food size and not by surface characteristics (taste) as postulated for other daphnid and some copepod species^49,50^. Studies by Burns^18^ indicate that the length of the carapax defines the upper size limit for ingestion (80 µm) and the mesh size of the filtering apparatus defines the lower limit (200 nm^51^). Assuming a non-selective uptake, the high capacity of *D. magna* to ingest small polystyrene beads but no 90 µm particles seems consistent and is in accordance with former studies on daphnids^23,24,52^. Swimming through the water column, the water flea encounters floating and settling particles. Owning this mobility we assume that *D. magna* preferably ingests suspended particles but is also able to feed on surficial sediment and neuston particles – resulting in a broad range of ingestible plastic materials.

### Additional particles

**Figure 2:**
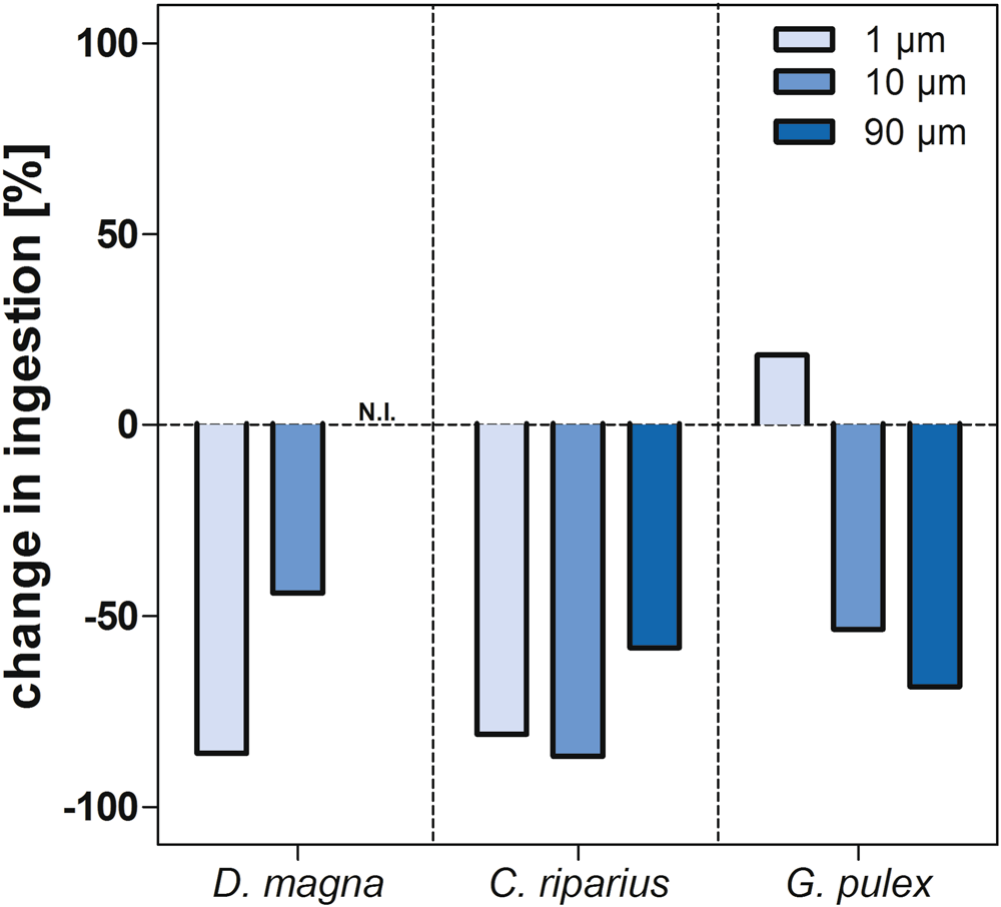
Influence of natural particles on the ingestion of polystyrene particles. Bar graphs show change in ingestion [%] in relation to the total amount of ingested particles per size (1, 10 and 90 µm) in the absence of additional particles. Negative values represent a reduced ingestion and positive values an enhanced ingestion of polystyrene beads in the presence of natural particles. N.I. = no ingestion.

Approaching more realistic exposure conditions and, in particular, to assess the ingestion of polystyrene beads within the framework of ecotoxicological test guidelines^53,54^, we co-exposed *D. magna*, *C. riparius* and *G. pulex* to microplastics and natural matter. The presence of algae significantly reduced the ingestion of 1 and 10 µm polystyrene spheres by *D. magna* (p < 0.05, Fig. 2, Supplementary Table S1 & S2). Similarly, the presence of sand reduced the uptake of the 1 (p < 0.01), 10 (p < 0.01) and 90 µm particles by *C. riparius*. In the presence of leaf material, *G. pulex* ingested less 10 and 90 µm polystyrene beads while the ingestion of 1 µm beads was increased by 18.3% (Fig. 2). This suggests a passive ingestion of 1 µm beads adhering to the leaf material. Additionally, in the egestion studies with *D. magna* and *C. riparius* we found that an exposure to food led to a shorter gut evacuation period of polystyrene spheres, confirming former studies on MP egestion^52^.

Furthermore, the presence of food decreased the amount of individuals with re-ingested particles (see Supplementary Information). Overall, the reduction of MP ingestion in the presence of other particles cannot be explained by the dilution alone. For instance, the ratio of PS beads to algae cells was 1:700 and to sand grains 1:35 at 3 000 P mL^−1^. In contrast, the ingestion of PS beads was only 7 times lower, suggesting a kind of selective feeding. However, since we did not use different concentrations of additional particles, the experimental design is not suitable to identify species-related selectivity. In general, the ability of freshwater invertebrates to select and preferably ingest food particles based on characteristics like size, shape and nutritional value is a well-known phenomenon (reviewed in Scherer *et al*.^55^). In theory, the species tested here are mainly categorized as generalist or omnivorous feeder in terms of their preferable food source. Thus, we hypothesize that the observed patterns are not affected by differences in the “nutritional value” of the offered natural particles (e.g., sand vs. algae).

In conclusion, a co-exposure to natural matter most commonly reduces the ingestion and enhances the egestion of polystyrene beads. This is important because it implies that species exposed to MPs alone have to cope with an increased number and duration of internal MP exposure compared to species which are exposed to MPs in the presence of food or natural particles.

### Development-dependent ingestion

Within our short-term experiments, we exposed specimens of a specific age to polystyrene spheres. To evaluate how the developmental stage affects the microplastics ingestion, we investigated the feeding on polystyrene spheres by different sizes and ages of the benthic feeder *C. riparius* and the pelagic feeder *D. magna*. Our results show that the size preferences as well as the amount of ingested polystyrene particles changes throughout the development.

**Figure 3:**
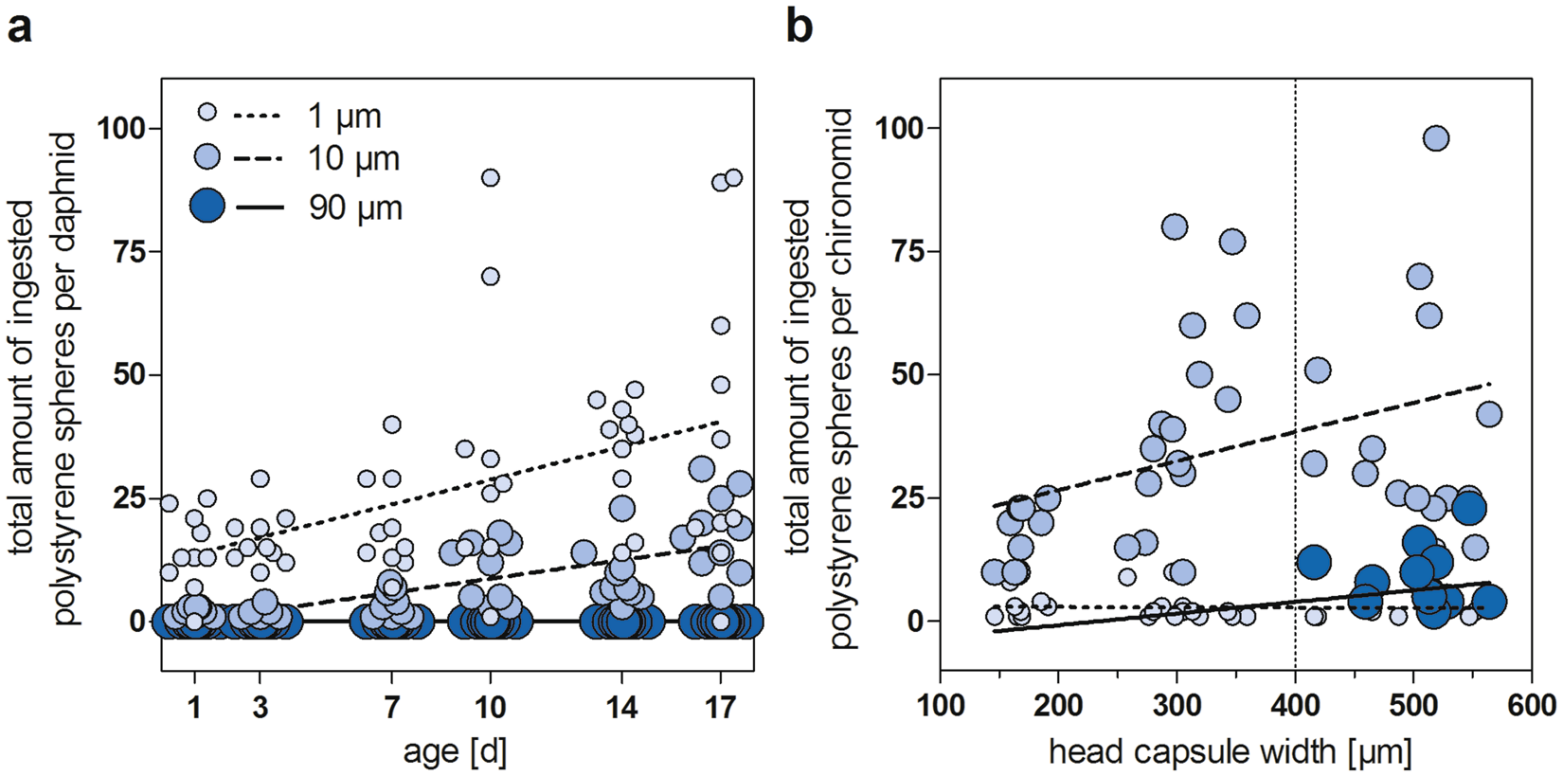
Development-dependent ingestion of polystyrene beads (1, 10 and 90 µm) by *Daphnia magna* (**a**) and the aquatic larvae of *Chironomus riparius* (**b**).

Regardless of the head capsule width (HCW) of *C. riparius*, the larvae ingested only a small amount of 1 µm polystyrene beads, while the amount of ingested 10 µm beads increased with an increasing size of the head capsule (Fig. 3b). 90 µm particles were solely detected in individuals with a HCW larger than 400 µm. This points to morphological restrictions by the size of the head capsule and functional mouth parts. Since L3 instars have HCWs of 260–400 µm^56^ and a corresponding mentum width of 62–96 µm (Supplementary Figure S3), we hypothesize that only L4 or large L3 larvae can ingest 90 µm particles. Although it is not possible to derive a maximum ingestible size based on our experimental data, large chironomid larvae with HCWs of 570 µm had mentum widths of 130 µm, which suggest that particles larger than 90 µm may be ingested.

*D. magna* did not ingest any 90 µm particles throughout their development, which is in accordance to the studies by Burns^18^. However, with an increasing age, the amount of 1 and 10 µm particles increased steadily (Fig. 3a). Comparative findings were reported by McMahon^32^ as feeding rates of *D. magna* on *Chlorella vulgaris* and *Saccharomyces cerevisiae* increased with increasing body size. Thus, the maximum ingestible size as well as the amount of ingested particles depends on the organism’s development, with the morphology (body size, exoskeleton and filtering capacities, Fig. 3, Supplementary Figure S3) determining the maximum ingestible size and the age-dependent physiology (feeding activity) determining the amount of ingested particles^18,32^.

### Implications for the environmental impact of microplastics

So far, data on the abundance of microplastics in freshwater is scarce^57–60^ with few studies reporting concentrations from 0.32 MP m^−3^ ^61^ to 12 932 MP m^−3^ ^62^ in the water column of large streams, and from 34 MP kg^−1^ ^57^ to 30 000 MP m^−2^ ^5^ in riverine sediments. In a worst case scenario, these concentrations are at least 25 times lower than in our ingestion studies. However, the particle sizes investigated here are normally not included in monitoring studies because they are too small to recover. Even so, some monitoring studies reported the presence of microplastics < 100 µm and polymer degradation studies indicate the formation of micro- to nano-sized polymer particles^6,63^. It is therefore likely that invertebrate species will encounter and ingest microplastics as demonstrated by recent fish monitoring studies^8^.

In general, feeding on particulate matter is quite common among primary consumers and thus, their capacity to ingest microplastics is not surprising. Toxicity studies with microplastics and invertebrates reported mainly adverse effects based on reduced dietary intake at high particle concentrations^14,25^. However, non-digestible matter is naturally occurring in aquatic ecosystems. Studies by Kirk and Gilbert^64^ documented a reduced population growth rate of cladocerans exposed to suspended clay (<2 µm), whereas more selective feeders like copepods were not affected. Therefore, one important question is whether microplastics behave differently than or induce different effects compared to other non-digestible particles. Here, depending on the polymers density and size, microplastics may be more available for pelagic filter feeders than sand or silt, which settle relatively fast. When a species cannot actively avoid microplastics intake, this might lead to a reduced food intake, inhibit population growth and support more selective feeders. The high capacity of *D. magna* to ingest plastic beads along with various other zooplankton species^20^ and mussels^65^ suggests that filter feeders are most prone to exposure to suspended microplastics.

### Conclusions

When evaluating the biological effects of microplastics in aquatic organisms, it is important to understand which abiotic and biotic factors determine their uptake and resulting internal exposure. We found that all investigated invertebrate species ingested polystyrene spheres in a concentration-dependent manner. The uptake rate was driven by size, which in turn depends on abiotic (sedimentation rates) as well as biotic (morphology, feeding strategies) factors. With regard to the latter, pelagic filter feeders appear to be specifically susceptible to microplastics ingestion. We also show that the presence of natural matter (food, sediment) significantly reduces the uptake of microplastics. Accordingly, the internal exposure will be lower in chronic toxicity studies and in natural habitats, where particulate matter is abundantly available. Finally, we demonstrated that the intake of microplastics depends on the invertebrates’ developmental stage with older specimens ingesting higher numbers and larger particles. Taken together, our study highlights the complex interactions between microplastics and biota, and how such interactions are influenced by abiotic and biotic factors. Although we solely tested freshwater invertebrates, we assume that the ingestion of microplastics by marine and estuarine invertebrates with similar autecology is affected by the investigated factors in our study. Now, the key issue is to determine whether exposure to microplastics result in effects that are different from naturally occurring matter.

## Methods

### Microplastic and natural particles

We used Fluoresbrite Yellow Green Microspheres (Polyscience Inc., Warrington, USA) with a density of 1.05 g cm^−3^ and a diameter of 1 µm, 10 µm and 90 µm, respectively, as model microplastics. The fluorescein-dyed microspheres (excitation: 441 nm, emission: 486 nm) were detectable using fluorescence microscopy (Olympus BX 50, BX-LA). The test concentrations of 3, 30, 300 and 3 000 P mL^−1^ were produced by diluting the purchased stock solutions with the corresponding culture medium (see below). To ensure a homogenous dispersion and an accurate serial dilution, the stock and prepared solutions were shaken and dispersed in ultrasonic bath (1 min) prior to each use. In case of the 90 µm beads, the highest tested concentration was 300 P mL^−1^ and the 3 and 30 P mL^−1^ concentrations were produced by transferring counted spheres to the test vessels. Unicellular green algae (*Scenedesmus obliquus*, in-house culture), quartz sand (Baumit, <700 µm) and leaf material (*Fagus sylvatica*, handpicked, conditioned for 7 days in natural stream water, cut to pieces of 0.25 cm^2^) were used as natural matter.

### Invertebrate species

To examine the capability of freshwater invertebrates to ingest microplastics, following species were used: The waterflea *Daphnia magna*, the aquatic larvae of the diptera *Chironomus riparius*, the freshwater snail *Physella acuta* and the blackworm *Lumbriculus variegatus* were obtained from in-house cultures with a light:dark cycle of 16:8 h. *D. magna* and *C. riparius* were cultured at 20 ± 1 °C in Elendt M4 medium according to OECD guideline 211^54^ and OECD guideline 219^53^, respectively. ISO medium^66^ was used for culturing *L. variegatus* (20 ± 1 °C) and *P. acuta* (25 ± 1 °C). The amphipod *Gammarus pulex* was collected from a small, local stream (Urselbach, Frankfurt am Main/Germany, 50°10'14.3“N 8°37'06.9“E) two weeks prior to experimental use and cultured in ISO medium at 16 ± 1 °C.

### Ingestion studies

The species were exposed individually (n = 6) to the three different microplastics sizes (1, 10 and 90 µm) and four different microplastics concentrations (3, 30, 300, 3 000 P mL^−1^) in 24-well plates. Before the start of the experiment, the organisms were transferred to freshly prepared medium (particle and food-free) for a 24 h depuration period. The test conditions were identical to the culture conditions and are in accordance with the respective OECD guidelines. The exposure duration (feeding period) and volume was optimized for each species according to the outcomes of a pilot ingestion and egestion study (see Supplementary Information for details): *D. magna* (6 d old): 2 min in 2 mL M4 medium, *C. riparius* (L3 larvae): 3 h in 2 mL M4 medium, *G. pulex* (10 mm length): 3 h in 2 mL ISO medium, *P. acuta* (38 d old): 24 h in 3 mL ISO medium, *L. variegatus* (7 d synchronized): 3 h in 2 mL ISO medium. The different exposure times reflected a compromise minimizing the inter-individual variations in feeding activity and excluding egestion of the microplastics to prevent an underestimation of feeding rates.

The experiments were terminated by washing the specimens of *D. magna* and *C. riparius* in de-ionized (DI) water followed by fixation in 70% ethanol. The individuals were mounted on slides and examined using fluorescence microscopy. Fluorescent microplastics particles in the gastrointestinal tract were counted. In case of *G. pulex* and *P. acuta*, it was not possible to analyze the intact organism because of their size and opaque tissue. Thus, they were washed with DI water to remove adhering microplastics, transferred to a reaction tube (1.5 mL, Eppendorf, Hamburg, Germany) with lysis buffer (100 mM Tris-HCl, 150 mM NaCl, 60 mM EDTA, 1% SDS, Proteinase K) and were homogenized with a pestle (disposable pestles and motor for pestles, VWR, Radnor, USA). The lysates were filtered (0.45 µm Metricel Black Membrane Filter, Pall Life Science) and examined using fluorescence microscopy. For *P. acuta* we observed the highest variability in short term exposure scenarios, mainly driven by extended resting periods of single individuals. Therefore, we exposed the snails over a longer period and counted the egested particles too (examination of faeces).

### Impact of natural particles on the feeding on microplastics

Natural particles like food or sand are integral components of chronic toxicity testing. To study their potential impact on the ingestion of polystyrene microplastics by 4 commonly used (ecotoxicological) model species, additional natural particles were included. In a parallel experiment (identical test conditions as described above), 2.1×10^6^ algae mL^−1^ (*Scenedesmus obliquus*) per daphnid (*D. magna*), 115 mg quartz sand (Baumit, <700 µm) per chironomid (*C. riparius*) and lumbriculid (*L. variegatus*) and 0.25 cm^2^ conditioned leaf fragments of *Fagus sylvatica* per amphipod (*G. pulex*) were applied.

### Microplastics ingestion throughout the development

In chronic toxicity studies, organisms are exposed over a longer period to assess potential effects throughout the life cycle. To test how the developmental stage is related to microplastic ingestion, we performed experiments with different life stages of *C. riparius* larvae and *D. magna*. For *C. riparius*, 40 randomly selected chironomids were obtained from the in-house culture and separated into four replicates, each containing 10 individuals and 50 mL M4 medium spiked with a mixture of 1, 10 and 90 µm beads at a concentration of 300 P mL^−1^ each. After a 3 h exposure the organisms were analyzed as described above. The head capsule width of the individuals (as proxy of the developmental stage) and the corresponding number and size of ingested particles was determined using ImageJ (Rasband, W.S., ImageJ). In addition, we analyzed the correlation between head capsule and mentum widths of 30 unexposed chironomid larvae (obtained from the in-house culture) to characterize morphological restrictions of the mouth parts (Supplementary Figure S3).

To evaluate the capacity to ingest polystyrene spheres throughout the development of *D. magna*, we exposed daphnids of different ages (1, 3, 7, 10, 14 and 17 d) to 1, 10 and 90 µm particles, respectively. In short term experiments, 10 daphnids of the same age were individually exposed to a mixture of 1, 10 and 90 µm fluorescent polystyrene beads with a concentration of 100 P mL^−1^ each. After a 2 min exposure, the daphnids were transferred into 70% ethanol, mounted on a slide and analyzed using fluorescent microscopy.

### Data analysis

Data were analyzed using GraphPad Prism 5 (GraphPad Software Inc.) and Statistica 12 (Statsoft Inc.). Surface plots (distance-weighted least squares, stiffness 0.25) were generated to detect patterns in the particle size preference. To compare the different exposure scenarios, we calculated ingestion rates using the variables P (amount of ingested particles) per t (exposure time in hours). The non-normal distribution and the heterogeneous variances of the data set required non-parametric analyzes. Therefore, we applied Mann-Whitney tests and Kruskal-Wallis tests with Dunn’s multiple comparison test (depending on the number of groups) to test for significant differences (p < 0.05) between the exposure scenarios. Here, we only included specimens ingesting microplastic particles. To detect significant influences of the variables size (1, 10 and 90 µm), additional natural particles (with and without) and species (*D. magna*, *C. riparius*, *G. pulex* and *P. acuta*) on the ingestion of microplastics we solely analyzed ingestion rates at an exposure to a microplastics concentration of 300 P mL^−1^.

## Electronic supplementary material

Supplementary Information
