# Supplementary material for: Feeding type and development drive the ingestion of microplastics by freshwater invertebrates
Source: Sci Rep. 2017 Dec 5;7:17006. doi: 10.1038/s41598-017-17191-7 (PMC5717137; doi:10.1038/s41598-017-17191-7)
Supplement: Supplementary file 1 — Supplementary Information [file 41598_2017_17191_MOESM1_ESM.pdf]

Supplementary Information

Feeding type and development drive the ingestion of microplastics by freshwater invertebrates

Authors

Christian Scherer[1], Nicole Brennholt[2], Georg Reifferscheid[2], Martin Wagner[1,3]

[1]Goethe University Frankfurt/ Main, Department Aquatic Ecotoxicology, Max-von-Laue-Strasse 13, D-60323 Frankfurt/ Main, Germany

[2]Federal Institute of Hydrology, Department Biochemistry and Ecotoxicology, Am Mainzer Tor 1, D-56002 Koblenz, Germany

[3]Norwegian University of Science and Technology, Department of Biology, Høgskoleringen 5, Realfagbygget, NO-7491 Trondheim, Norway.

Corresponding Author

E-mail: c.scherer@bio.uni-frankfurt.de

Ingestion studies

Table S1: Results of the non-parametric analysis of the ingestion rates for *Daphnia magna*, *Chironomus riparius*, *Gammarus pulex* and *Physella acuta*. Significant influences (n.s. = not significant, ★ = $p < 0.05$, ★★ = $p < 0.01$, ★★★ = $p < 0.001$) of the variables size (1, 10 and 90 µm), additional natural particles (with and without) and organisms were solely analysed for ingestion rates at 300 P mL^{-1}.

				D. magna	***C. riparius***	***G. pulex***	***P. acuta***
	Add. particles	Size [µm]	Analysed c [P mL^{-1}]	Significant differences	Significant differences	Significant differences	Significant differences
Concen-tration	Without (-)	1	3	n.s.	n.s.	n.s.	*vs* 3000★★★
			30	*vs* 3000 ★★★	*vs* 3000 ★★	*vs* 3000 ★	*vs* 3000 ★
			300	n.s.	*vs* 3000 ★	n.s.	*vs* 3★
			3000	n.s.	n.s.	n.s.	n.s.
	Without (-)	10	3	n.s.	n.s.	n.s.	*vs* 3000 ★★★
			30	*vs* 3000 ★★★	*vs* 3000 ★★★	n.s.	*vs* 3000 ★★
			300	n.s.	n.s.	*vs* 3000 ★	n.s.
			3000	n.s.	n.s.	n.s.	n.s.
	Without (-)	90	3	n.s.	n.s.	n.s.	*vs* 300★
			30	n.s.	n.s.	n.s.	n.s.
			300	n.s.	n.s.	n.s.	n.s.
	With (+)	1	3	n.s.	n.s.	n.s.	-/-
			30	n.s.	*vs* 3000 ★★	*vs* 3000 ★★	-/-
			300	n.s.	*vs* 3000 ★	n.s.	-/-
			3000	n.s.	n.s.	n.s.	-/-
	With (+)	10	3	n.s.	n.s.	n.s.	-/-
			30	n.s.	n.s.	n.s.	-/-
			300	n.s.	n.s.	n.s.	-/-
			3000	n.s.	n.s.	n.s.	-/-
	With (+)	90	3	n.s.	n.s.	n.s.	-/-
			30	n.s.	n.s.	n.s.	-/-
			300	n.s.	n.s.	n.s.	-/-
Add. particles	(-) *vs* (+)	1	300	★	★★	n.s.	-/-.
	(-) *vs* (+)	10	300	★★	★★	n.s	-/-
	(-) *vs* (+)	90	300	n.s.	n.s.	n.s	-/-
Size	Without (-)	1 *vs* 10 *vs* 90	300	n.s.	10 *vs* 90 ★★	n.s.	1 *vs* 90 ★
	With (+)	1 *vs* 10 *vs* 90	300	1 *vs* 10★	1 *vs* 10 ★★	n.s	-/-
Organism	Without (-)	1	300	*G. pulex* ★★★<br>*P. acuta* ★	n.s.	*D. magna* ★★★	*D. magna* ★
	Without (-)	10	300	*G. pulex* ★★★	*G. pulex*★	*C. riparius*★<br>*D. magna*★★★	n.s.
	Without (-)	90	300	n.s.	*P. acuta*★	n.s.	*C. riparius*★
	With (+)	1	300	*C. riparius*★★	*D. magna*★★	n.s.	n.s.
	With (+)	10	300	*G. pulex* ★★	n.s.	*D. magna* ★★	n.s.
	With (+)	90	300	n.s.	n.s.	n.s.	n.s.

Table S2: Results of the ingestion studies with *Daphnia magna*, *Chironomus riparius*, *Gammarus pulex* and *Physella acuta*. Mean ± standard deviation (ingested particles), min/ max values for ingested particles (min; max) and number of bead containing specimens (specs. cont. p, %).

**Without additional particles (-)**		**1 µm**				**10 µm**				**90 µm**		
		3 [P mL^{-1}]	30 [P mL^{-1}]	300 [P mL^{-1}]	3000 [P mL^{-1}]	3 [P mL^{-1}]	30 [P mL^{-1}]	300 [P mL^{-1}]	3000 [P mL^{-1}]	3 [P mL^{-1}]	30 [P mL^{-1}]	300 [P mL^{-1}]
Daphnia magna	ing. particles:	0 P	4.6 ± 2.6 P	31.6 ± 18.1 P	147.6 ± 26.6 P	1 ± 0.62 P	4.17 ± 2.98 P	26.5 ± 7.45 P	152.3 ± 35.4 P	0 P	0 P	0 P
	specs. cont. P:	0 %	100%	100%	100 %	83.3%	100%	100%	100%	0 %	0 %	0 %
	min; max:	0; 0	3; 10	3; 51	120; 197	0; 2	2; 10	15; 36	103; 206	0; 0	0; 0	0; 0
Chironomus riparius	ing. particles:	0.17 ± 0.41 P	0.3 ± 0.51 P	15.7 ± 10.24 P	47 ± 12.9 P	1.17 ± 1.17 P	10.3 ± 12.1 P	95.8 ± 38.2 P	531.2 ± 110.4 P	0.5 ± 1.21 P	0.5 ± 0.84 P	1 ± 0.88 P
	specs. cont. P:	16.6%	33.3%	100%	100%	66.6%	100%	100%	100%	16.6%	33.3%	66.6%
	min; max:	0; 1	0; 1	6; 32	39; 62	0; 3	4; 35	53; 156	418; 680	0; 3	0; 2	0; 2
Gammarus pulex	ing. particles	0 P	0.67 ± 0.82 P	3.17 ± 1.82 P	6.17 ± 2.23 P	0 P	0 P	3 ± 4.1 P	13.17 ± 11.9 P	0.16 ± 0.41 P	3.33 ± 3.43 P	8.17 ± 11.9 P
	specs. cont. P:	0%	50%	100%	100%	0%	0%	100 %	100%	16.6%	83.3%	100%
	min; max:	0; 0	0; 2	1; 6	3; 9	0; 0	0; 0	1; 12	8; 36	0; 1	0; 10	1; 32
Physella acuta	ing. particles	19.2 ± 6.21 P	66.2 ± 20.4 P	86.2 ± 16.2 P	830 ± 145 P	36.2 ± 19.1 P	62 ± 27.2 P	459 ± 73.6 P	2496 ± 247 P	4 ± 0.88 P	37.5 ± 14.5 P	641 ± 174 P
	specs. cont. P:	100%	100%	100%	100%	100%	100%	100%	100%	100%	100%	100%
	min; max:	10; 29	43; 104	72; 113	671; 993	13; 64	34; 114	393; 594	2079; 2841	3; 5	25; 56	376; 816
Lumbriculus variegatus	ing. particles	0 P	0 P	0.83 ± 2.04 P	0 P	0 P	1.16 ± 2.85 P	3 ± 9.79 P	0 P	0.3 ± 0.81 P	0 P	0 P
	specs. cont. P:	0 %	0 %	16.6%	0 %	0 %	16.6%	16.6%	0 %	16.6%	0 %	0 %
	min; max:	0; 0	0; 0	0; 5	0; 0	0; 0	0; 7	0; 24	0; 0	0; 2	0; 0	0; 0

**With additional particles (+)**		**1 µm**				**10 µm**				**90 µm**		
		3 [P mL^{-1}]	30 [P mL^{-1}]	300 [P mL^{-1}]	3000 [P mL^{-1}]	3 [P mL^{-1}]	30 [P mL^{-1}]	300 [P mL^{-1}]	3000 [P mL^{-1}]	3 [P mL^{-1}]	30 [P mL^{-1}]	300 [P mL^{-1}]
Daphnia magna	ing. particles:	0.16 ± 0.41 P	0.6 ± 0.82 P	4.33 ± 3.01 P	20.5 ± 12.63 P	0.3 ± 0.52 P	2 ± 1.66 P	10.83 ± 6.40 P	89.3 ± 48.1 P	0 P	0 P	0 P
	specs. cont. P:	16.6 %	50%	100%	100 %	33.3%	83.3%	100%	100%	0 %	0 %	0 %
**+ algae**	min; max:	0; 1	0; 2	2; 10	3; 40	0; 1	0; 4	3; 20	30; 151	0; 0	0; 0	0; 0
Chironomus riparius	ing. particles:	0 P	0.5 ± 0.55 P	1.6 ± 0.82 P	9.82 ± 5.42 P	0 P	1.5 ± 1.86 P	14.5 ± 7.64 P	68.3 ± 31.67 P	0 P	0.16 ± 0.41 P	0.67 ± 1.62 P
	specs. cont. P:	0%	50%	100%	100%	0%	66.6%	100%	100%	0%	16.6%	16.6%
**+ sand**	min; max:	0; 0	0; 1	1; 3	5; 20	0; 0	0; 5	3; 23	35; 122	0; 0	0; 1	0; 4
Gammarus pulex	ing. particles	0 P	0.83 ± 0.74 P	3.67 ± 1.21 P	7.3 ± 4.93 P	0 P	0 P	1.3 ± 1.37 P	6.67 ± 6.22 P	0.17 ± 0.41 P	1.82 ± 3.53 P	1.67 ± 3.13 P
	specs. cont. P:	0%	66.6%	100%	100%	0%	0%	66.6 %	100%	16.6%	50%	50%
**+ leaf**	min; max:	0; 0	0; 2	2; 5	3; 14	0; 0	0; 0	0; 3	1; 16	0; 1	0; 9	0; 8
Lumbriculus variegatus	ing. particles	0 P	0 P	0 P	3.33 ± 8.15 P	0.66 ± 1.62 P	0 P	0 P	5.5 ± 13.46 P	0 P	0.3 ± 0.82 P	0.16 P
	specs. cont. P:	0 %	0 %	0%	16.6%	16.6 %	0%	0%	16.6 %	0%	16.6 %	16.6 %
**+ sand**	min; max:	0; 0	0; 0	0; 0	0; 20	0; 4	0; 0	0; 0	0; 33	0; 0	0; 2	0; 1

Egestion experiments

Material and Methods

Daphnia magna. For every time interval (t_0-t_{11}) and bead size (1 and 10 µm), we exposed 5 daphnids per replicate (n = 5) to 300 P mL^{-1} fluorescent polystyrene spheres. After a 5 min exposure, the daphnids were washed for 1 min by transferring them to particle-free M4 medium. The daphnids were then transferred to fresh 50 mL M4 medium. After t_1 = 2 min, t_2 = 4 min, t_3 = 8 min, t_4 = 16 min, t_5 = 32 min, t_6 = 64 min, t_7 = 128 min, t_8 = 256 min, t_9 = 512 min, t_{10} = 1 440 min and t_{11} = 2 880 min we removed the daphnids and sacrificed them for microscopic examination. The ingestion of polystyrene spheres was verified by analysing the gut content of t_0 daphnids. To evaluate the impact of algae on the gut evacuation period of microplastics, we added 10^6 cells mL^{-1} per treatment. In a second study, we exposed daphnids to 10 µm polystyrene particles (300 P mL^{-1}) and documented the evacuation process via live imaging. For this, after a feeding period of 5 min, 10 daphnids were washed and transferred individually in multiwall plates containing fresh M4 medium and analysed using fluorescence microscopy after 2, 6, 14, 30, 60, 126 and 254 min.

Chironomus riparius. We exposed aquatic larvae of *C. riparius* to 10 µm (3000 P mL^{-1}) and 90 µm (300 P mL^{-1}) polystyrene spheres each. After a 3 h exposure period, the larvae were examined using fluorescent microscopy. Only larvae that ingested microplastics were used for the egestion studies. To prevent re-ingestion of egested particles, we transferred them individually (n = 6) to cages made of stainless steel gauze (mesh size of 0.5 mm). These cages were placed into 100 mL beakers filled with 50 mL M4 medium. Thus, faeces could pass the mesh and sink to the bottom of the beakers, while the larvae remained in the cages. After 0.5, 1, 1.5, 2, 2.5, 3, 12, 24 and 48 h the larvae were examined using fluorescent microscopy. Specimens containing polystyrene spheres were transferred back into the cages. To evaluate

the impact of food on the gut evacuation period of microplastics, we added a tip of a spatula of ground TetraMin fish food.

Results

Daphnids exposed to algae during the egestion period, completely egested the polystyrene beads within 128 min for the 10 µm spheres and 1 140 min for 1 µm polystyrene spheres (Fig.S1 a-b). This point to a slower egestion of 1 µm compared to 10 µm particles. In the absence of algae cells, the amount of bead containing individuals remained steady (Fig. S1 a-b). Re-ingestion of egested particles as well as slower gut evacuation processes seems likely. Observations and real-time tracking via live imaging support these assumptions (Fig. S1 c-d). Without algae, the gut clearance was slowed down and a re-ingestion of egested particles was observed frequently.

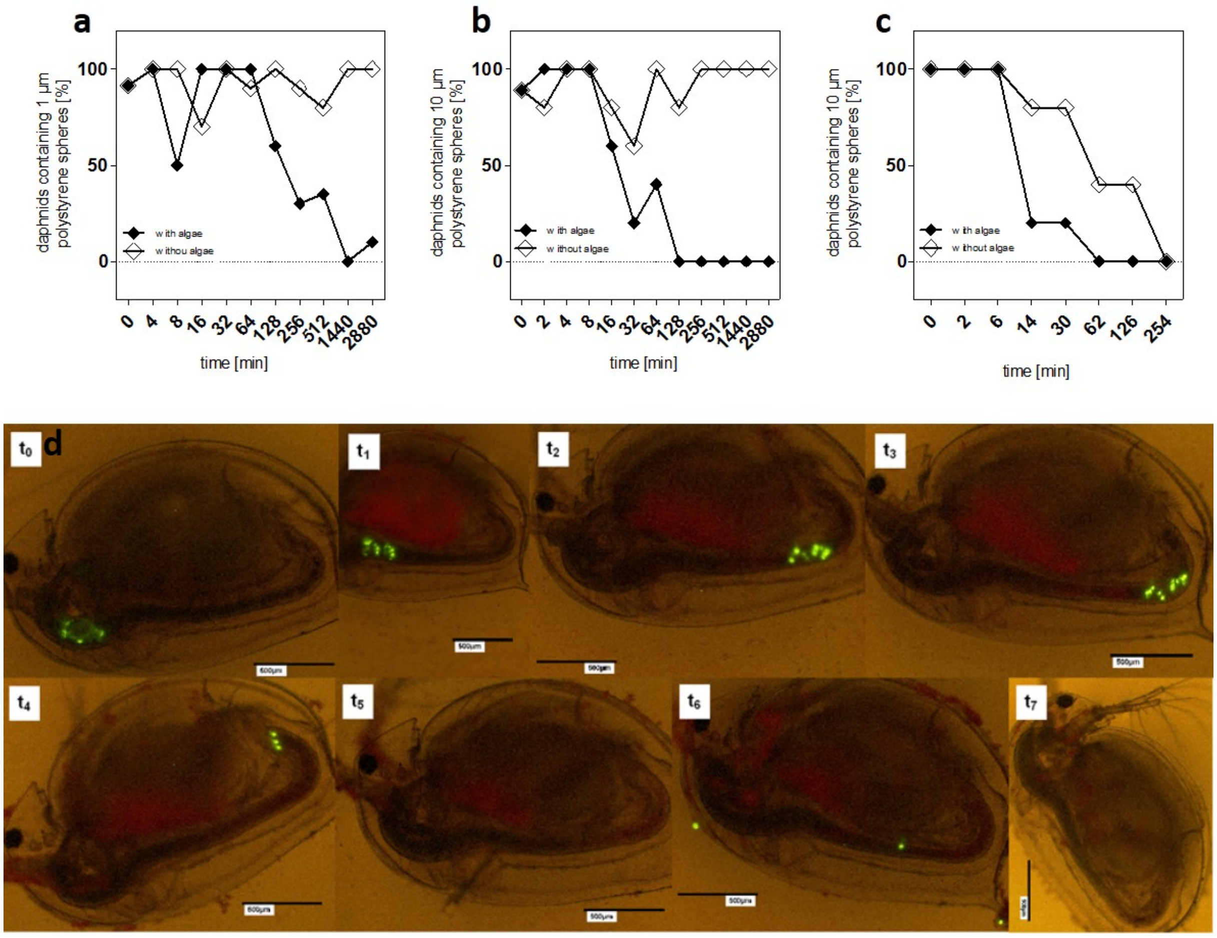

Figure S1: Egestion experiment with *Daphnia magna* and fluorescent polystyrene beads. (a-c) Ratio of daphnids containing beads in their gastrointestinal tract (%) at specific time points for 1 µm beads (a) and 10 µm beads (b-c). The results from the live imaging (c, d). Re-ingested particles (d, t_6) were excluded in (c).

In the egestion experiments with *C. riparius*, larvae co-exposed to food had a slightly faster gut clearance compared to individuals exposed to microplastics only (Fig. S2). Within the first 3 h the larvae only excreted a few beads located at the near end of the gastrointestinal tract. A complete clearance was not been observed. Overall, 90 µm polystyrene beads were egested faster than 10 µm beads. However, despite the cages, we cannot completely exclude a re-ingestion of faeces.

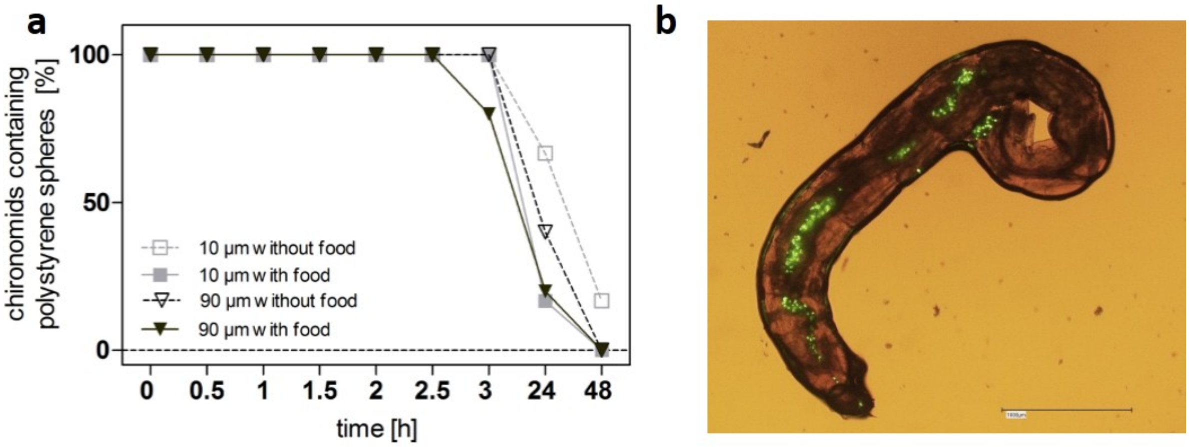

Figure S2: Egestion experiment with aquatic larvae of *Chironomus riparius* and fluorescent polystyrene spheres. (a) Ratio of larvae containing 10 and 90 µm beads at specific time points in the presence and absence of food. (b) Larva after 3 h depuration (10 µm).

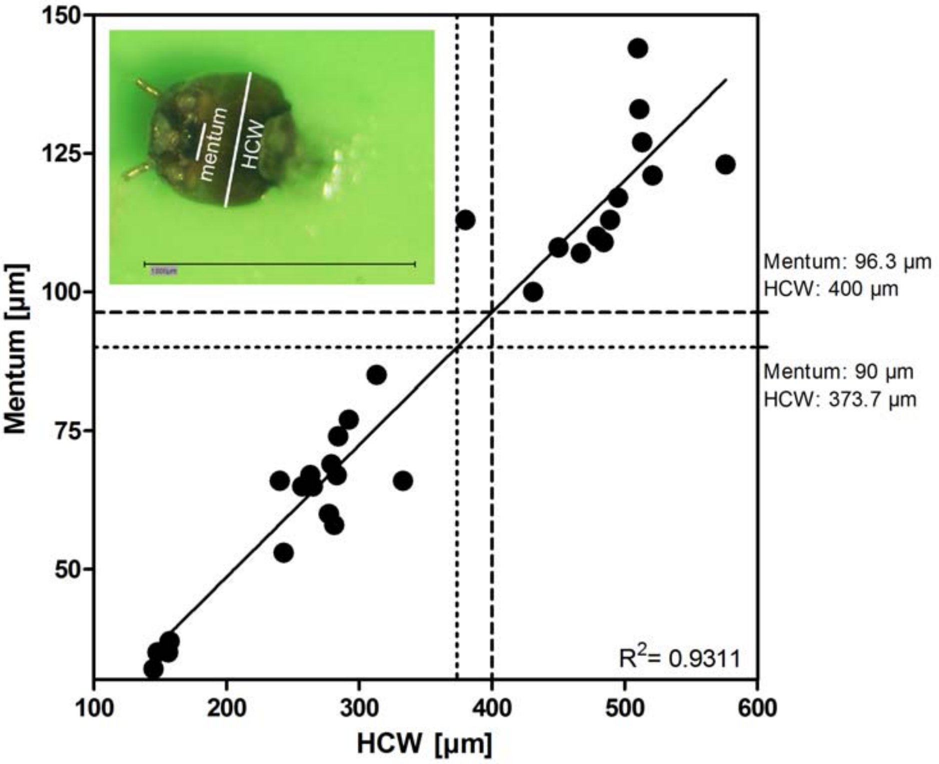

Figure S3: Correlation (r^2= 0.9311) of head capsule (HCW) and mentum width of 30 analysed *Chironomus riparius* larvae (unexposed controls). Dotted lines represent morphological restrictions, whereas only individuals with HCWs >400 µm and mentum width >96.3 µm have the theoretically capacity to ingest 90 µm polystyrene spheres.
